# Analysis of Risk Scoring for Placenta Accreta Spectrum and Its Impact on Maternal and Fetal Outcomes

**DOI:** 10.7759/cureus.95208

**Published:** 2025-10-23

**Authors:** Komal P Raut, Sornam MS, Jasmine Kavitha Washington

**Affiliations:** 1 Obstetrics and Gynecology, Sree Balaji Medical College and Hospital, Chennai, IND

**Keywords:** fetal outcome, high-risk pregnancy, maternal outcomes, placenta accreta spectrum, risk profile

## Abstract

Background: Placenta accreta spectrum (PAS) disorders, characterized by abnormal adherence or invasion of the placenta into the myometrium, are associated with significant maternal and neonatal morbidity. Early identification of high-risk cases is essential to optimize delivery planning and reduce adverse outcomes. The Placenta Accreta Index (PAI), integrating clinical history and ultrasound parameters, has been proposed as a predictive tool for PAS. This study aimed to evaluate the diagnostic accuracy of the PAI in predicting PAS and its utility in anticipating maternal and neonatal outcomes.

Materials and methods: A prospective observational study was conducted on 150 pregnant women at risk for PAS. A single experienced sonographer performed all ultrasound evaluations and PAI scoring to minimize observer bias. The PAI incorporated the number of prior cesarean sections, placental lacunae grade, sagittal myometrial thickness, anterior placenta previa, and bridging vessels. A standardized management protocol was followed: antenatal corticosteroids for fetal lung maturity, magnesium sulfate for neuroprotection when <34 weeks, and multidisciplinary team involvement (obstetricians, anesthetists, neonatologists, and blood bank). MRI or Doppler studies were used selectively to confirm placental invasion depth, and delivery timing (34-36 weeks or earlier for antepartum bleeding) was guided by imaging and clinical stability. Intraoperative findings and histopathological examination (HPE) served as reference standards. Maternal and neonatal outcomes were recorded.

Results: Among 150 participants, the placenta was posterior in 84 (56%), anterior in 33 (22%), low-lying in 18 (12%), and fundal in 15 (10%). Placental lacunae were most commonly Grade 2 (49, 32.7%), and myometrial thickness <1 mm was observed in 58 (38.7%). Placenta previa was present in 105 (70%) women. HPE findings revealed a normal placenta in 142 (94.7%), absence of decidua with thin Nitabuch’s layer in 4 (2.7%), thin Nitabuch’s layer in 3 (2%), and villi directly contacting myometrium in 1 (0.7%). The PAI demonstrated sensitivity 100% (95% CI: 2.5-100), specificity 97.3% (95% CI: 92.8-99.2), positive predictive value 20% (95% CI: 3.6-62.4), negative predictive value 100%(95% CI: 97.4-100), and overall diagnostic accuracy 97.3% (95% CI: 92.9-99.2). Maternal outcomes were favourable in 145 (96.7%) cases; one woman (0.7%) required hysterectomy, and four (2.7%) had excessive bleeding managed conservatively.

Conclusions: PAI is a reliable, non-invasive tool for identifying women at high risk for PAS. High PAI scores can guide prenatal counselling, delivery planning, and resource allocation, while low scores effectively rule out severe placental invasion, supporting safer conservative management. The use of PAI may have the potential to improve maternal and neonatal outcomes in pregnancies complicated by PAS.

## Introduction

Placenta accreta spectrum (PAS) disorders, also referred to as abnormal invasive placenta, represent a group of pathological conditions in which trophoblastic tissue infiltrates abnormally beyond the decidua basalis and into the uterine myometrium. In severe forms, the invasion can progress through the uterine serosa and extend to adjacent pelvic organs. Based on the depth and extent of this invasion, PAS is subclassified into three distinct entities: placenta accreta (when the invasion involves less than 50% of the myometrium), placenta increta (when the invasion exceeds 50% of the myometrial thickness), and placenta percreta (when the placenta penetrates completely through the uterine wall and potentially involves nearby organs such as the bladder). This abnormal placentation is primarily attributed to defective decidualization, a physiological process that normally limits trophoblastic invasion. When this mechanism fails, the anchoring villi of the placenta infiltrate excessively into the myometrium, giving rise to PAS disorders [1].

Clinically, PAS is of paramount importance due to the life-threatening hemorrhage it may provoke. Attempting manual separation of an abnormally adherent placenta during delivery often results in uncontrollable bleeding, exposing mothers to catastrophic consequences. The burden of PAS has been rising worldwide, largely in parallel with the increasing frequency of cesarean section deliveries [2]. Epidemiological data show significant variation in incidence across populations. In India, the reported incidence of PAS disorders is approximately 0.9 per 1000 births, while in China, figures are as high as 0.22% of all births, considering national data, underscoring its growing public health relevance [3]. Currently, PAS is recognized as a leading contributor to maternal morbidity and, in severe cases, maternal mortality.

The complications associated with PAS stem primarily from massive obstetric hemorrhage. Blood loss during delivery may be so extensive that it leads to hypovolemic shock, metabolic acidosis, and coagulopathy. The management of such cases often requires large-volume blood transfusions, which carry their own risks, including transfusion-related reactions and infections. Women with PAS may also require prolonged intensive care support, with some progressing to multi-organ dysfunction and acute kidney injury. At its worst, maternal death remains a possible outcome [4-7]. Despite these grim prospects, timely antenatal diagnosis of PAS has been shown to significantly mitigate adverse outcomes, allowing clinicians to plan delivery in tertiary centers with multidisciplinary expertise, adequate blood bank facilities, and access to interventional radiology and critical care support.

Advances in prenatal imaging have made it possible to identify PAS during pregnancy. Ultrasonography is widely accepted as the first-line modality due to its accessibility, non-invasiveness, and ability to be repeated at multiple stages of gestation. Several ultrasound signs, including loss of the retroplacental clear zone, thinning of the myometrium, placental lacunae, and bridging vessels, are considered indicative of PAS. However, ultrasound is not without its limitations. Diagnostic accuracy may be influenced by operator experience, maternal body habitus, and particularly the location of the placenta, with posteriorly implanted placentae posing greater diagnostic challenges [6].

To overcome these limitations, magnetic resonance imaging (MRI) has been employed as a supplementary tool for the diagnosis and staging of PAS. MRI provides superior soft tissue contrast and a wider field of view, allowing better assessment of invasion depth and extra-uterine spread [7,8]. Nevertheless, routine use of MRI remains controversial due to its limited availability, high cost, need for advanced expertise, and the frequent use of gadolinium-based contrast agents, which continue to raise concerns regarding fetal safety [9]. Consequently, MRI is generally reserved for complex or equivocal cases, particularly when posterior placenta previa or suspicion of placenta percreta is present.

Recognizing the limitations of imaging modalities alone, researchers have developed composite diagnostic scoring systems to improve prediction accuracy for PAS. One such system is the Placenta Previa with Adherent Placenta score, which incorporates maternal history, ultrasound features, and MRI findings to estimate PAS risk [10]. Although promising, subsequent studies have indicated that MRI does not always contribute significant additional diagnostic value beyond ultrasound and may be warranted only in selective scenarios, such as posteriorly located placentae or highly suspected percreta cases [11,12].

More recently, the Placenta Accreta Index (PAI) has been introduced as a clinically applicable risk-scoring model for PAS. The PAI integrates both clinical and sonographic parameters, including history of previous cesarean delivery, placental location, thinnest sagittal myometrial thickness, presence of intraplacental lacunae, and detection of bridging vessels. Each parameter is assigned a weighted score, and the cumulative total (ranging from 0 to 9) provides an estimated probability of PAS [13,14]. Studies evaluating the PAI have shown encouraging diagnostic performance, with good predictive accuracy for differentiating PAS from non-PAS cases. The significance of the PAI has been recognized internationally. It has been incorporated into practice guidelines by both the International Federation of Gynaecology and Obstetrics and the American College of Obstetricians and Gynecologists [15,16].

Given the rising incidence of PAS, its devastating maternal-fetal consequences, and the evolving landscape of diagnostic approaches, there is a clear need for validated, evidence-based risk assessment tools. Against this backdrop, the present study was designed to assess the diagnostic accuracy and predictive value of PAI in identifying PAS disorders. In addition, the study aims to evaluate how the application of such risk scoring impacts maternal and fetal outcomes. By doing so, we seek to provide further clarity on the role of structured scoring systems in improving antenatal diagnosis, guiding delivery planning, and ultimately reducing the burden of morbidity and mortality associated with PAS.

## Materials and methods

Study design and objectives

This was a hospital-based observational study designed to evaluate the diagnostic value of PAI in predicting PAS disorders and associated maternal and neonatal outcomes. The PAI was calculated using ultrasound parameters and relevant obstetric history, aiming to determine whether a composite score could reliably identify women at high risk for morbidly adherent placenta. In addition to diagnostic performance, the study assessed maternal and neonatal complications associated with PAS and explored the implications of the PAI in delivery planning and patient counselling.

Study population and setting

The study included a total of 150 pregnant women, all of whom were suspected to be at risk of PAS based on ultrasound findings and obstetric history. Patients were recruited using a consecutive sampling technique, ensuring that every eligible woman had an equal chance of inclusion. All women underwent routine antenatal care and follow-up at the study hospital, which also served as the site for diagnostic evaluation, delivery, and management of PAS cases.

Sample size and calculation

Assuming an expected sensitivity and specificity of 0.95, a PAS prevalence of 5.3% based on prior literature [16], a 95% confidence level (Z = 1.96), and a desired absolute precision (L) of ±0.07, the estimated total sample size required to achieve adequate precision around sensitivity was approximately 701, compared to 40 for specificity; thus, the larger value (701) would govern. Allowing a wider margin of error (L = 0.10) would reduce the required sample to about 343 participants. In practice, 150 women were enrolled for feasibility, yielding eight PAS-positive cases.

Calculation of the Placenta Accreta Index

PAI was derived by assigning weighted values to specific ultrasound parameters and obstetric history factors, with the maximum total score being 9. The parameters included in the index were selected based on established diagnostic predictors of PAS.

A history of two or more previous lower-segment caesarean sections was assigned a score of 3.0, reflecting the well-documented association between repeat caesarean deliveries and abnormal placentation. Placental lacunae, which represent irregular vascular spaces within the placenta observed on ultrasound, were graded and weighted accordingly: Grade 3 lacunae were assigned 3.5 points, while Grade 2 lacunae were assigned 1.0 points. Sagittal myometrial thickness was another key parameter, as thinning of the uterine wall is a known indicator of invasive placentation. A thickness less than 1 mm was scored as 1.0, a thickness between 1 and 3 mm as 0.5, and 3-5 mm as 0.25. Anterior placenta previa contributed 1.0 points, while the presence of bridging vessels, which represent abnormal vascular connections crossing the placental-uterine interface, was given a score of 0.5. The total PAI score for each patient was obtained by summing the individual values. Based on prior validation studies, a score greater than 5 was considered to indicate a high probability of invasive placentation [17].

Timing of delivery and intraoperative assessment

Women with suspected PAS based on their PAI score and ultrasound findings were planned for elective cesarean section between 34 and 36 weeks of gestation. However, if an antepartum hemorrhage occurred, the decision for earlier intervention was made to safeguard maternal and fetal health. During cesarean delivery, intraoperative findings were meticulously documented. Particular attention was paid to evidence of abnormal placental adherence, such as difficulty in placental separation, excessive bleeding, or invasion into surrounding tissues.

In cases where a cesarean hysterectomy was required due to morbid adherence, the surgical specimens were subjected to histopathological examination (HPE). Histopathology served as the gold standard for confirming the diagnosis of PAS and was used to validate the clinical and ultrasound-based findings. HPE was performed for all surgically managed and suspected PAS cases where tissue was available.

Maternal outcomes

The study comprehensively documented maternal complications associated with PAS and their correlation with PAI scores. Postpartum hemorrhage (PPH) was one of the primary outcomes assessed, given its strong link with invasive placentation. Other maternal morbidities included hemorrhagic shock, disseminated intravascular coagulation, bladder injury, sepsis, and the requirement for intensive care unit (ICU) admission. Maternal mortality was also recorded, although emphasis was placed on morbidity as a marker of disease burden. These outcomes were compared between women with high PAI scores (>5) and those with lower scores to evaluate the index’s predictive ability for clinical severity.

Neonatal outcomes

In addition to maternal complications, neonatal outcomes were systematically analyzed. Adverse outcomes included low birth weight, intrauterine death, and the need for neonatal ICU (NICU) admission. These outcomes provided insight into the perinatal implications of PAS and the impact of early planned delivery on neonatal health. The relationship between maternal PAI scores and neonatal outcomes was explored to assess whether higher maternal risk translated into poorer neonatal prognosis.

Management strategies

The management of PAS in the study population was individualized based on intraoperative findings and the degree of placental invasion. Three main approaches were documented: cesarean hysterectomy, expectant management, and conservative management. Caesarean hysterectomy was the definitive management for women with clinically significant invasion, especially when massive bleeding or maternal instability was encountered. In select cases with limited invasion and stable hemodynamics, expectant or conservative approaches were attempted to preserve the uterus and fertility. The choice of management was correlated with the PAI score to explore whether the index could guide preoperative planning.

Management categories

Caesarean Hysterectomy

This approach was performed in women with high PAI scores (>5), anterior placenta previa, or intraoperative evidence of deep invasion or uncontrollable bleeding. The placenta was left in situ, and the hysterectomy was completed without attempting removal. Adjuncts included internal iliac or aortic balloon occlusion, ureteric stenting, and stepwise devascularization.

Expectant Management

This approach was used when the patient was hemodynamically stable, bleeding was minimal, and fertility preservation was desired. The placenta was left in situ after fetal delivery, the cord ligated close to insertion, and the uterus closed without removal. Follow-up included serial ultrasound and Doppler monitoring; uterine artery embolization was available for secondary hemorrhage.

Conservative Management

This approach was applied for focal accreta with limited invasion and adequate hemostasis. Only the adherent placental segment was excised, and the uterine defect was repaired to preserve fertility. Local compression sutures, uterotonics, or selective vessel ligation were used as adjuncts.

Outcome Comparison

Maternal and neonatal outcomes were compared between PAI ≤5 and PAI >5 groups to assess whether higher PAI scores correlated with increased rates of hysterectomy, transfusion, blood loss, and NICU admission.

Ethical considerations

This study was conducted in compliance with the Declaration of Helsinki (2013 revision) and received prior approval from the Institutional Ethics Committee of Sree Balaji Medical College and Hospital (approval number: 002/SBMCH/IHEC/2023/2036). Written informed consent was obtained from all participants for inclusion of anonymized clinical data and ultrasound or intraoperative images. Patient confidentiality was maintained through de-identification and coded data handling, ensuring that no personal identifiers were retained in the final dataset. Safety monitoring for intraoperative hemorrhage and transfusion-related events was performed by the attending obstetrician and anesthesiology team under a predefined protocol, which included continuous hemodynamic assessment, real-time blood loss quantification, and activation of a massive transfusion protocol if required. The study imposed no financial burden, ensured respect for patient autonomy, and aimed to generate evidence to improve diagnosis, counselling, and management of PAS in future practice.

Statistical analysis

All collected data were subjected to rigorous statistical analysis using SPSS Statistics version 24.0 (IBM Corp. Released 2016. IBM SPSS Statistics for Windows, Version 24.0. Armonk, NY: IBM Corp.). Categorical variables, such as presence or absence of lacunae or bridging vessels, were presented as frequencies and percentages. Continuous variables, such as maternal age and myometrial thickness, were expressed as means with standard deviations. The normality of distribution was confirmed before performing appropriate statistical tests.

The diagnostic accuracy of the PAI was evaluated through sensitivity, specificity, positive predictive value (PPV), and negative predictive value (NPV) calculations at different score thresholds. A PAI score greater than 5 was considered high risk, and outcomes were analyzed accordingly. Results were displayed in both tabular and graphical forms to facilitate clear visualization and interpretation. In all analyses, a p-value of less than 0.05 was considered statistically significant.

## Results

In this study of 150 women, the majority were aged 26-30 years (120, 80%), followed by 21-25 years (18, 12%) and 31-35 years (12, 8%). The mean age was 28 ± 2.05 years. Most participants had a marriage duration of 1-3 years (136, 90.67%), with only 13 (8.67%) married for 4-6 years and 1 (0.66%) for 7-9 years. Socioeconomically, 129 (86%) belonged to the lower middle class, while 21 (14%) were from the upper middle class. All women had booked cases (100%). The most common presenting complaint was spotting per vaginum (5, 3.33%), while pain in the lower abdomen was reported by two (1.33%). Regarding parity, 70 (46.67%) were multiparous, 57 (38%) nulliparous, and 23 (15.33%) primiparous. Abortion history was present in 82 (54.67%) and absent in 68 (45.33%). Based on BMI, the majority were pre-obese (102, 68%), followed by obese class I (47, 31.33%). Only one (0.67%) had a normal BMI, with a mean BMI of 28.6 ± 1.9. Among comorbidities, previous LSCS was the most frequent (91, 60.67%), followed by hypertension (63, 42%) and diabetes mellitus (26, 17.33%) (Table 1).

**Table 1:** Sociodemographic characteristics of study participants (n = 150) SD: standard deviation, PV: per vaginam, LSCS: lower segment cesarean section * modified BG Prasad scale [18]

Variable	Categories/values	n (%)
Age group	21–25 years	18 (12)
	26–30 years	120 (80)
	31–35 years	12 (8)
	Mean ± SD	28 ± 2.05
Marriage duration	1–3 years	136 (90.67)
	4–6 years	13 (8.67)
	7–9 years	1 (0.66)
Socioeconomic status	Upper middle	21 (14)
	Lower middle	129 (86)
Booking status	Booked	150 (100)
	Unbooked	0 (0)
Presenting complaints	Pain lower abdomen	2 (1.33)
	Spotting PV	5 (3.33)
Parity	Nullipara	57 (38)
	Primipara	23 (15.33)
	Multipara	70 (46.67)
Abortion history	Present	82 (54.67)
	Absent	68 (45.33)
Body mass index class	18.0–24.99 (normal)	1 (0.67)
	25.0–29.99 (pre-obese)	102 (68)
	30.0–34.99 (obese I)	47 (31.33)
	Mean ± SD	28.6 ± 1.9
Comorbidity	Previous LSCS	91 (60.67)
	Diabetes mellitus	26 (17.33)
	Hypertension	63 (42)

In the present study of 150 women, the placenta was most commonly located posteriorly in 84 (56%) cases, followed by anterior in 33 (22%), low-lying in 18 (12%), and fundal in 15 (10%). Regarding lacunar grading, Grade 2 was the most frequent finding (49, 32.67%), followed by Grade 1 in 43 (28.67%), Grade 3 in 32 (21.33%), and Grade 0 in 26 (17.33%). Myometrial thickness assessment revealed that 58 (38.67%) women had <1 mm thickness, 57 (38%) had >3 mm, and 35 (23.33%) had 1-3 mm thickness. Placental bulge was observed in 22 (14.67%) participants, while absent in 128 (85.33%). Placenta previa was present in 105 (70%) cases, whereas 45 (30%) showed no evidence of previa (Table 2).

**Table 2:** Ultrasonographic characteristics of placenta in the study population (n = 150)

Variable	Categories/findings	n (%)
Placental location	Anterior	33 (22)
	Posterior	84 (56)
	Fundal	15 (10)
	Low lying	18 (12)
Grade of lacunae	Grade 0	26 (17.33)
	Grade 1	43 (28.67)
	Grade 2	49 (32.67)
	Grade 3	32 (21.33)
Myometrial thickness	>3 mm	57 (38)
	1–3 mm	35 (23.33)
	<1 mm	58 (38.67)
Placental bulge	Present	22 (14.67)
	Absent	128 (85.33)
Placenta previa	Present	105 (70)
	Absent	45 (30)

**Figure 1:**
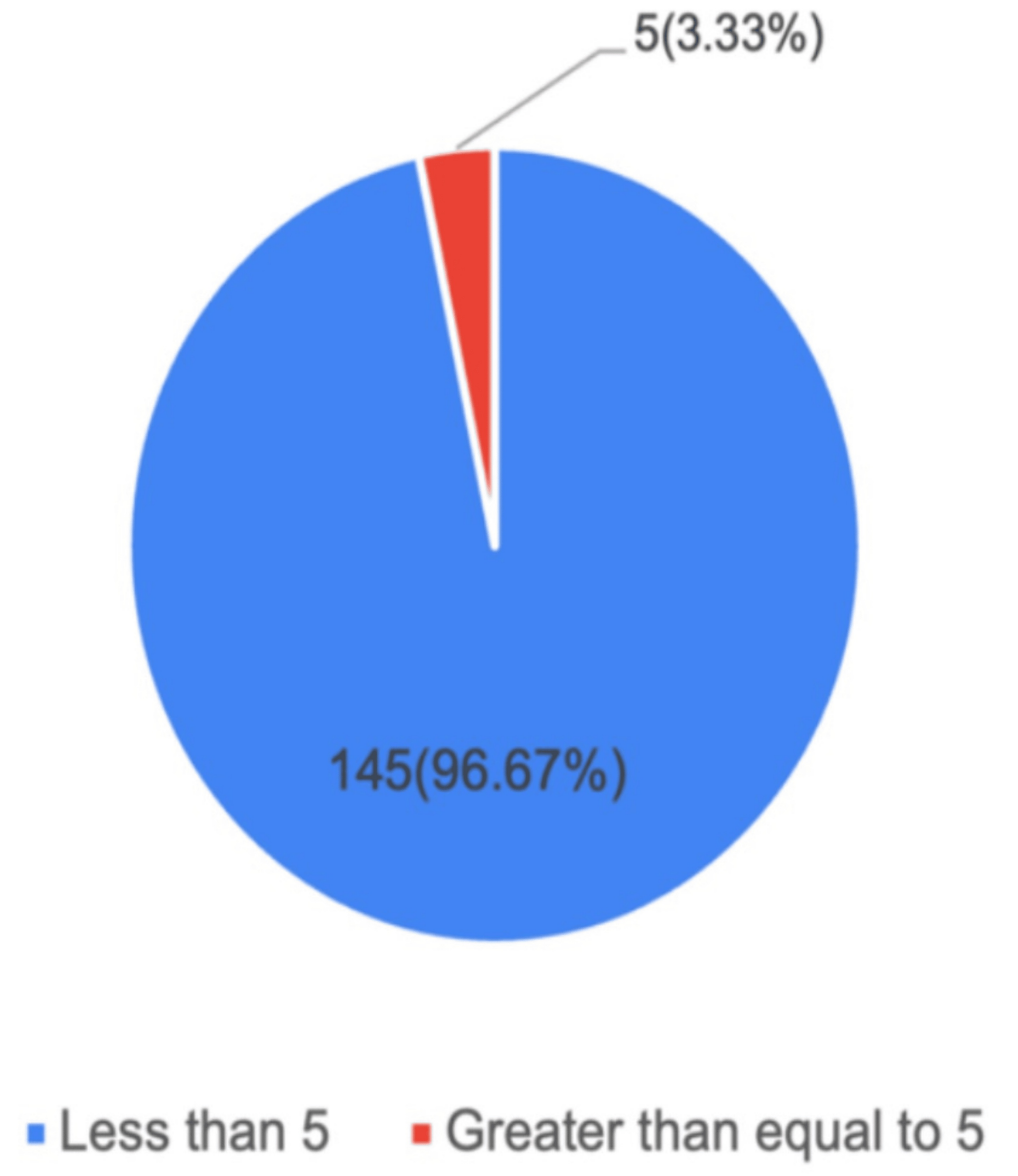
Distribution of cases according to PAI score among study participants (n = 150) This figure shows the distribution of cases according to the PAI score among study participants (n = 150). Of these, 145 (96.67%) cases had a PAI score of <5, indicating a low risk of placental invasion, while the remaining 5 (3.33%) cases had a PAI score >5. PAI: Placenta Accreta Index

In the present study, 145 (96.67%) cases have a PAI score <5, indicating a low risk of placental invasion, while the remaining five (3.33%) cases have a score >5, indicating a high risk. Figure 1 depicts the distribution of cases according to the PAI score.

HPE of the placental specimens in the study population (n = 150) revealed that the majority of cases were normal, accounting for 142 (94.67%). Abnormal findings indicative of PAS were observed in a small proportion of cases, including absence of decidua with a thin Nitabuch’s layer in 4 (2.67%), a thin Nitabuch’s layer in 3 (2%), and villi in direct contact with myometrial muscle without an intervening decidua in 1 (0.66%). These findings highlight that while most placentas appeared histologically normal, a minority demonstrated pathological features consistent with abnormal placental adherence (Table 3).

**Table 3:** HPE findings of the placenta in the study population (n = 150) HPE: histopathological examination

HPE findings	No.	%
Normal	142	94.67
Absence of decidua with a thin Nitabuch's layer	04	2.67
Thin Nitabuch's layer	03	02
Villi are in direct contact with the myometrial muscle without an intervening decidua	01	0.66

The diagnostic performance of the PAI score was evaluated against HPE findings in the study population. The PAI demonstrated a sensitivity of 100% (95% CI: 2.5-100), indicating that all true cases of placenta accreta were correctly identified. Specificity was 97.3% (95% CI: 92.8-99.2), reflecting the high ability of the score to correctly identify cases without accreta. PPV was 20% (95% CI: 3.6-62.4), while NPV was 100% (95% CI: 97.4-100), demonstrating that a low PAI score reliably ruled out the presence of placenta accreta. Overall diagnostic accuracy was 97.3% (95% CI: 92.9-99.2) (Table 4).

**Table 4:** Diagnostic performance of the PAI score against HPE findings (n = 150) PAI: Placenta Accreta Index, HPE: histopathological examination, PPV: positive predictive value, NPV: negative predictive value

Parameter	Value (%)	95% CI
Sensitivity	100.0	2.5–100.0
Specificity	97.3	92.8–99.2
PPV	20.0	3.6–62.4
NPV	100.0	97.4–100.0
Diagnostic accuracy	97.3	92.9–99.2

In this study, maternal outcomes were generally favorable. The majority of women, 145 (96.67%), and their babies were reported to be well without any significant complications assessed within six weeks. One woman (0.67%) required a hysterectomy due to PPH. In four cases (2.66%), excessive bleeding occurred but was managed conservatively, with the mothers remaining hemodynamically stable; however, their babies required NICU admission due to respiratory distress syndrome (RDS) (Table 5).

**Table 5:** Distribution of cases according to maternal and neonatal outcome (n = 150) PPH: postpartum hemorrhage, RDS: respiratory distress syndrome, NICU: neonatal intensive care unit

Maternal outcome	No.	%
Hysterectomy due to PPH	01	0.67
Mother and baby are both well	145	96.67
Excessive bleeding managed conservatively, mother hemodynamically stable, baby in RDS in NICU	04	2.66

## Discussion

This study provides a comprehensive analysis of the application of PAI in a cohort of 150 pregnant women, predominantly characterized by a high prevalence of risk factors for PAS, notably a previous cesarean section (60.67%) and placenta previa (70%). The central finding of this investigation is the robust diagnostic performance of the PAI, which demonstrated a sensitivity and NPV of 100%, alongside a high specificity (97.3%) and overall accuracy (97.3%). These results affirm the critical role of a standardized ultrasound-based scoring system in the prenatal identification and risk stratification of PAS, a condition whose management is profoundly dependent on accurate antepartum diagnosis.

The perfect sensitivity (100%) and NPV (100%) of the PAI in our cohort are its most significant attributes. This indicates that in our patient population, no case of PAS confirmed on histopathology was missed by the PAI score. This is paramount for clinical practice, as a false-negative result could lead to an unprepared delivery team facing catastrophic, life-threatening hemorrhage. The high NPV provides immense reassurance; a low PAI score effectively rules out PAS, potentially allowing for a trial of labor or a less complex surgical approach in selected cases, thereby avoiding unnecessary, radical surgeries in women without the condition. This finding aligns with the objective of prenatal screening for PAS, which is to achieve the highest possible sensitivity to prevent unforeseen morbidity.

However, the relatively low PPV of 20% warrants careful interpretation. This means that for every five women flagged as "high-risk" by the PAI, only one had histopathological confirmation of PAS. While this may seem suboptimal, it is a common statistical phenomenon in populations where the disease prevalence is low. The histopathological incidence of PAS in our study was low (5.33%, 8/150), which inherently limits the PPV. From a clinical safety perspective, a low PPV is often considered acceptable for a condition like PAS, as the consequences of missing the diagnosis are far greater than the consequences of a false positive. A false-positive PAI score triggers a management protocol that involves planning delivery at a tertiary care center with a multidisciplinary team (MDT) and ensuring blood bank availability. These precautions are beneficial for any complex case of placenta previa, regardless of the final histological diagnosis. Our results are consistent with the study by Zhang et al. (2023), who also developed an ultrasound scoring system and emphasized the critical importance of high sensitivity, even at the cost of a moderate PPV, to ensure patient safety [19].

The ultrasound findings in our study population reflect a high-risk cohort. The high frequency of lacunar grade 2 or 3 (54%) and myometrial thickness <1 mm (38.67%) is a well-established sonographic marker of invasive placentation. These features are integral components of the PAI and other scoring systems because they directly correlate with the underlying pathology: the disruption of the normal uteroplacental interface. The HPE findings, though abnormal in only a small subset, corroborate the ultrasound suspicions. The observed abnormalities, such as absence of decidua, a thin Nitabuch’s layer, and villi in direct contact with myometrium, are the hallmarks of PAS, confirming the failure of normal decidualization in the area of a uterine scar.

Our demographic data further contextualizes the risk. The cohort had a mean age of 28 years and a mean BMI in the pre-obese range (28.6 kg/m²), which aligns with known epidemiological trends. Yang et al. (2023) identified advanced maternal age and high BMI as significant risk factors that can exacerbate the prognosis of PAS [20]. Furthermore, the very high rate of previous LSCS (60.67%) is the single most important etiological factor for PAS, as the cesarean scar creates a niche where decidualization is defective, facilitating abnormal trophoblast invasion. This is compounded by the high prevalence of placenta previa (70%), a combination that dramatically increases the risk of PAS, as extensively documented in the literature. The retrospective analysis by Varlas et al. (2021) strongly emphasized that the confluence of placenta previa and a prior uterine scar should be considered the primary indicator for rigorous PAS screening [21].

The overwhelmingly favorable maternal outcomes in this study, with 96.67% of women and their babies well, with a hysterectomy rate of only 0.67%, are a testament to the success of a protocol that includes systematic prenatal ultrasound screening. The four cases (2.66%) of conservatively managed excessive bleeding highlight that even when PAS is suspected or confirmed, radical surgery is not always necessary. With an MDT approach, techniques such as uterine artery embolization or conservative surgery (e.g., focal resection) can be attempted, preserving fertility.

The need for NICU admission for babies born to mothers with excessive bleeding due to RDS underscores the iatrogenic prematurity that is a recognized consequence of planned preterm delivery in PAS cases. This reinforces the recommendation that delivery planning by an MDT should include neonatologists to optimize neonatal outcomes. Our excellent outcomes contrast with historical cohorts where PAS was often diagnosed intraoperatively, leading to higher rates of massive transfusion, hysterectomy, and ureteral or bowel injury. This comparison strongly supports the value of prenatal diagnosis and planned delivery, as advocated by Zhou et al. (2023), who highlighted that clinical indicators combined with diagnostic tools significantly improve prognosis [22]. This suggests a future where multimodal approaches (ultrasound, MRI, and serum markers) might further refine risk prediction. Furthermore, while our study focused on singleton pregnancies, the principles of PAS risk assessment also apply to multiple gestations. However, the epidemiology may differ, as noted by Guo et al. (2022) in their analysis of a Chinese population [23].

A few limitations must be acknowledged. First, this was a single-center study, which may limit the generalizability of our findings to populations with different demographics, referral patterns, and obstetric practices. Second, the low absolute number of histologically confirmed PAS cases (n = 8) limits the precision of our sensitivity and PPV estimates, as reflected by the wide confidence intervals (e.g., PPV 95% CI: 3.6-62.4). Third, the study did not fully adjust for potential confounders, such as prior uterine surgeries other than cesarean section, maternal comorbidities, or variations in the timing of ultrasound evaluation, all of which could influence both PAI scores and clinical outcomes. Additionally, the ultrasound grading of placental invasion is operator-dependent. Although a single experienced sonographer performed all scans to reduce inter-observer variation, some degree of subjectivity remains inherent to image interpretation. Finally, as this study was conducted in a tertiary referral center, there is a possibility of selection bias toward higher-risk pregnancies, potentially overestimating disease prevalence and diagnostic accuracy compared to general obstetric populations.

The findings of this study have important implications for clinical practice. The PAI provides a practical, noninvasive, and reproducible tool to identify women at high risk for clinically significant PAS, enabling better prenatal planning and individualized management. High PAI scores can guide obstetricians to anticipate potential complications, such as massive PPH, and prepare for interventions, including elective cesarean hysterectomy, availability of blood products, and ICU support. Conversely, a low PAI score reliably rules out severe placental invasion, allowing for safer expectant or conservative management. The index can also facilitate structured patient counselling regarding maternal and fetal risks, timing of delivery, and the likelihood of surgical intervention. Incorporating the PAI into routine prenatal assessment may ultimately improve maternal and neonatal outcomes by reducing emergency interventions, optimizing resource allocation, and enhancing multidisciplinary preparedness for high-risk deliveries.

## Conclusions

This study demonstrates that PAI is an exceptionally sensitive tool for the prenatal detection of PAS in a high-risk population. Its perfect NPV makes it an invaluable rule-out test, while its application leads to improved maternal and neonatal outcomes through meticulous multidisciplinary planning. Low PPV, a function of the disease's rarity, underscores that a high PAI score should be viewed as a mandate for preparation rather than a definitive diagnosis. Future research should focus on prospective, multi-institutional validation of the PAI and the exploration of integrating serum biomarkers to further enhance predictive accuracy, ultimately aiming to optimize care for every pregnancy complicated by this challenging condition.
